# Supplementary material for: Complexome profiling of the Chlamydomonas psb28 mutant reveals TEF5 as an early PSII assembly factor
Source: Plant Cell. 2025 Mar 17;37(6):koaf055. doi: 10.1093/plcell/koaf055 (PMC12164590; doi:10.1093/plcell/koaf055)
Supplement: koaf055_Supplementary_Data [file koaf055_supplementary_data.zip › psb28_interactive.zip!/plots/intensity/qualityPlots/peptides/PSBO_Cre09.g396213.t1.1.html]

### Cre09.g396213.t1.1

Trivial name: PSBO  
SeqCoverage [median]: 61.90  
Mass: 30.58 kDa  
  
 

| Sequence | MissedCleavage | Mass [Da] | wtIntensity | muIntensity | Score |
| --- | --- | --- | --- | --- | --- |
| ADAEELLK | 0 | 887.46 | 147934.87 | 32679.53 | 205.13 |
| ADAEELLKENVK | 1 | 1357.71 | 1611546.17 | 322811.80 | 202.27 |
| ADAEELLKENVKITK | 2 | 1699.94 | 29707.66 | 37077.65 | 95.35 |
| ALKGSAVFSVAK | 1 | 1176.69 | 224891.84 | 31590.87 | 143.42 |
| EDDGIDYAATTVQLPGGER | 0 | 2005.92 | 24229.41 | 4562.33 | 181.77 |
| EESQFK | 0 | 766.35 | 145342.11 | 31727.24 | 140.01 |
| EESQFKGGETEFVK | 1 | 1613.76 | 282985.48 | 42580.05 | 152.99 |
| EESQFKGGETEFVKTK | 2 | 1842.90 | 1345.98 | 1698.49 | 44.57 |
| GDFLVPSYR | 0 | 1052.53 | 958704.66 | 363622.82 | 155.88 |
| GGETEFVK | 0 | 865.42 | 467089.11 | 186758.43 | 219.74 |
| GGETEFVKTK | 1 | 1094.56 | 20342.72 | 9407.28 | 143.96 |
| GGSTGYDNAVALPAR | 0 | 1447.71 | 1022461.38 | 209988.29 | 305.20 |
| GGSTGYDNAVALPARADAEELLK | 1 | 2317.16 | 33056.88 | 6730.77 | 124.63 |
| GGSTGYDNAVALPARADAEELLKENVK | 2 | 2787.40 | 179354.93 | 56754.88 | 110.68 |
| GRGGSTGYDNAVALPAR | 1 | 1660.83 | 571286.07 | 121805.40 | 152.71 |
| GRGGSTGYDNAVALPARADAEELLK | 2 | 2530.28 | 4740.96 | 0.00 | 42.45 |
| GRGGSTGYDNAVALPARADAEELLKENVK | 3 | 3000.53 | 113409.69 | 19238.73 | 91.60 |
| GSAVFSVAK | 0 | 864.47 | 1073411.91 | 119184.09 | 194.22 |
| GSAVFSVAKVDPVTGEIAGVFESIQPSDTDLGAKPPK | 2 | 3712.92 | 1731.26 | 0.00 | 38.99 |
| GSAVFSVAKVDPVTGEIAGVFESIQPSDTDLGAKPPKDIK | 3 | 4069.13 | 2313.15 | 0.00 | 77.35 |
| GSSFLDPK | 0 | 849.42 | 1253378.96 | 221498.17 | 166.68 |
| GSSFLDPKGR | 1 | 1062.55 | 924.23 | 3608.39 | 82.42 |
| GTLDNIK | 0 | 759.41 | 364043.59 | 109352.94 | 216.16 |
| GTLDNIKGDFLVPSYR | 1 | 1793.93 | 405090.07 | 164555.63 | 174.40 |
| LTYTLDAMSGSFK | 0 | 1432.69 | 1645388.21 | 671316.11 | 291.24 |
| QFDGKGTLDNIK | 1 | 1334.68 | 261778.19 | 20710.61 | 112.02 |
| QFDGKGTLDNIKGDFLVPSYR | 2 | 2369.20 | 71063.97 | 5602.42 | 80.99 |
| VAFLFTIK | 0 | 937.56 | 1198263.50 | 564600.90 | 186.67 |
| VAFLFTIKQFDGK | 1 | 1512.83 | 7109.50 | 0.00 | 71.24 |
| VAFLFTIKQFDGKGTLDNIK | 2 | 2254.24 | 6273.83 | 0.00 | 77.39 |
| VDPVTGEIAGVFESIQPSDTDLGAKPPK | 1 | 2866.46 | 106940.32 | 0.00 | 147.45 |
| VDPVTGEIAGVFESIQPSDTDLGAKPPKDIK | 2 | 3222.67 | 185138.65 | 0.00 | 124.97 |
| VGSDGSAELK | 0 | 961.47 | 80935.17 | 6112.70 | 161.28 |
| VGSDGSAELKEDDGIDYAATTVQLPGGER | 1 | 2949.38 | 295827.05 | 19792.56 | 195.30 |
| VTGLWYAQLK | 0 | 1177.65 | 752515.02 | 79014.62 | 224.70 |
|  |  |  | 13550556.5 | 3464383.7 |  |
